# Deficiency of FLCN in Mouse Kidney Led to Development of Polycystic Kidneys and Renal Neoplasia

**DOI:** 10.1371/journal.pone.0003581

**Published:** 2008-10-30

**Authors:** Jindong Chen, Kunihiko Futami, David Petillo, Jun Peng, Pengfei Wang, Jared Knol, Yan Li, Sok-Kean Khoo, Dan Huang, Chao-Nan Qian, Ping Zhao, Karl Dykyma, Racheal Zhang, Brian Cao, Ximing J. Yang, Kyle Furge, Bart O. Williams, Bin Tean Teh

**Affiliations:** 1 Laboratory of Cancer Genetics, Van Andel Research Institute, Grand Rapids, Michigan, United States of America; 2 Course of Applied Marine Biosciences, Graduate School of Marine Science and Technology, Tokyo University of Marine Science and Technology, Konan, Minato-ku, Tokyo, Japan; 3 Genomic Medicine Institute, Cleveland Clinic, Cleveland, Ohio, United States of America; 4 Stowers Institute for Medical Research, Kansas City, Missouri, United States of America; 5 Laboratory of Cell Signaling and Cancinogenesis, Van Andel Research Institute, Grand Rapids, Michigan, United States of America; 6 Laboratory of Germline Modification and Cytogenetics, Van Andel Research Institute, Grand Rapids, Michigan, United States of America; 7 Sun Yat-Sen University Cancer Center, Guangzhou, Guangdong, China; 8 Laboratory of Antibody Technology, Van Andel Research Institute, Grand Rapids, Michigan, United States of America; 9 Laboratory of Computational Biology, Van Andel Research Institute, Grand Rapids, Michigan, United States of America; 10 Surgical Pathology, Northwestern University Feinberg School of Medicine, Feinberg, Chicago, Illinois, United States of America; 11 NCCS-VARI Translational Cancer Research Laboratory, National Cancer Centre, Singapore; Stanford University, United States of America

## Abstract

The Birt–Hogg–Dubé (BHD) disease is a genetic cancer syndrome. The responsible gene, *BHD*, has been identified by positional cloning and thought to be a novel tumor suppressor gene. *BHD* mutations cause many types of diseases including renal cell carcinomas, fibrofolliculomas, spontaneous pneumothorax, lung cysts, and colonic polyps/cancers. By combining Gateway Technology with the *Ksp-Cre* gene knockout system, we have developed a kidney-specific *BHD* knockout mouse model. *BHD^flox/flox^/Ksp-Cre* mice developed enlarged kidneys characterized by polycystic kidneys, hyperplasia, and cystic renal cell carcinoma. The affected *BHD^flox/flox^/Ksp-Cre* mice died of renal failure at approximate three weeks of age, having blood urea nitrogen levels over tenfold higher than those of *BHD ^flox/+^/Ksp-Cre* and wild-type littermate controls. We further demonstrated that these phenotypes were caused by inactivation of *BHD* and subsequent activation of the mTOR pathway. Application of rapamycin, which inhibits mTOR activity, to the affected mice led to extended survival and inhibited further progression of cystogenesis. These results provide a correlation of kidney-targeted gene inactivation with renal carcinoma, and they suggest that the *BHD* product FLCN, functioning as a cyst and tumor suppressor, like other hamartoma syndrome–related proteins such as PTEN, LKB1, and TSC1/2, is a component of the mTOR pathway, constituting a novel FLCN-mTOR signaling branch that regulates cell growth/proliferation.

## Introduction

Birt–Hogg–Dubé (BHD) syndrome is an autosomal dominant genetic disease characterized by fibrofolliculomas (follicular hamartomas), renal cell carcinomas, spontaneous pneumothorax, and lung cysts[1]. Renal cysts were also observed in some patients[2], [3]. The *BHD* gene (accession number, BC015687), located on chromosome 17p11.2, contains 14 exons spanning approximately 20 kb of genomic DNA and encodes a protein of 579 amino acids, folliculin (FLCN) that has no known functional domains [4], [5], [6]. Germ-line mutations, somatic alterations, and loss of *BHD* mRNA have been observed in patients with BHD, colorectal cancer, and in some cases of gastric cancer; thus, *BHD* may be viewed as a candidate tumor-suppressor gene[7], [8], [9], [10]. Germ-line mutations of the counterpart *BHD* have also been identified in dogs and rats having renal cell carcinomas and renal multiple cysts [11], [12], [13].

As one of the hamartoma syndromes, BHD shares many clinical features (such as follicular hamartomas, mucosal fibromas, and internal malignancy) with Cowden syndrome (CD, affected gene *PTEN*), Peutz-Jeghers syndrome (PJS, affected gene *LKB1*), and tuberous sclerosis complex (affected genes *TSC1/TSC2*) [14], [15], [16]. Of these, Cowden syndrome shares the most clinical features with BHD. While PTEN, LKB1, and TSC1/2 are critical members of the mTOR pathway [17], the *BHD* protein FLCN has also been suggested to be involved [18], [19]. These findings imply that FLCN, like PTEN, may also be a pivotal tumor suppressor gene and a potential player in mTOR pathway. Over the last few years, interest in FLCN has grown significantly. A few model organisms have been used to explore the physiological role of FLCN. However, these studies presented discrepant results, which leave the function of FLCN elusive. In *Drosophila*, the Bhd homologue was linked to JAK-STAT and Dpp pathway[20]. An *in vitro* experiment revealed that FLCN interacts with AMPK in mammalian cell lines, associating FLCN with the mTOR pathway[18], whereas in fission yeast, Bhd was reported to activate the mTOR counterpart Tor2, presenting an opposite role to Tsc1/2[19].

Since no *in vitro* experiments or nonmammalian model can replicate the complex processes of tumorigenesis in humans, the development of *BHD*-deficient animal models will shed light on the role of *BHD in vivo* and on the *BHD*-related biochemical pathways responsible for neoplasia, which eventually could lead to the development of therapeutic agents against *BHD*-related diseases. Although natural mutants could be used for experimental models, the possibilities of homozygous embryonic lethality and additional unknown genetic changes often impede further analysis of the phenotypes and the physiological role of the gene. The genetically engineered conditional knockout mouse model can bypass this barrier and provide a “cleaner” and more versatile system for functional studies of *BHD* gene protein FLCN. While it might be a suppressor of mouse cystogenesis demonstrated by a recent study[21], *BHD* is expected to be a potential tumor suppressor gene whose mutations have led to renal tumors and other diseases in BHD patients. Therefore, it is essential to further elucidate whether kidney-specific knockout of *BHD* in the mouse is also implicated in kidney tumorigenesis, and what mechanism is involved.

## Results

### Generation of *BHD* conditional knockout construct and mice

**Figure 1:**
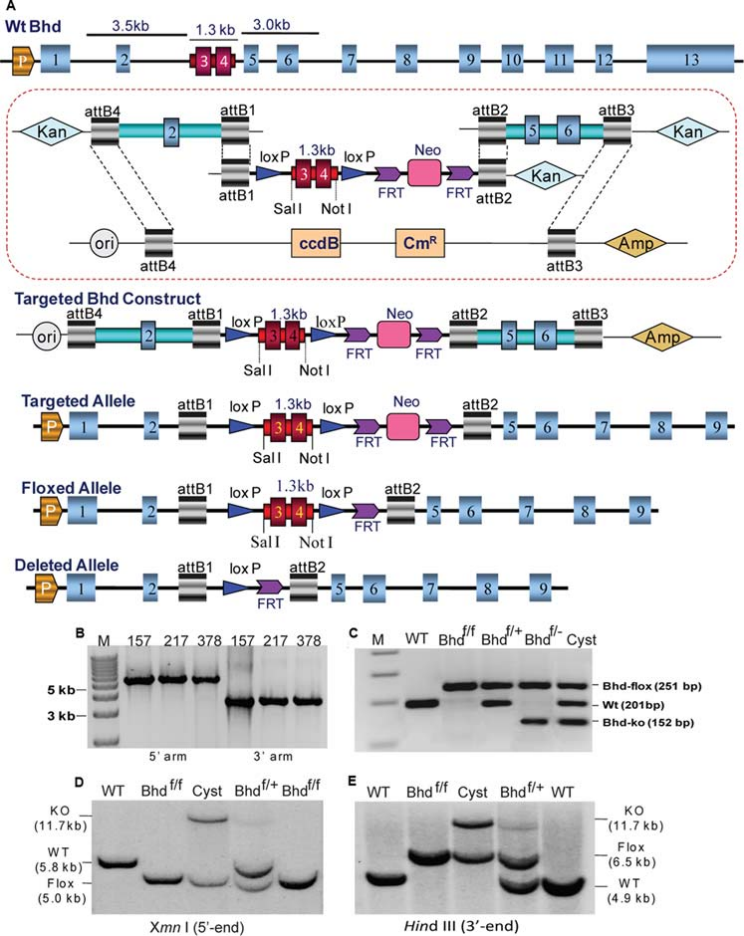
Targeting strategy and generation of *BHD* conditional knockout mice. (A) Construction of the *BHD* gene targeting vector using a combination of the Gateway and *loxp* systems. A 3.5-kb 5′ homology arm containing exon 2 and a 3.0-kb 3′ arm carrying exons 5 and 6 were integrated into the pDONR P4-P1R and pDONR P2R-P through a BP (attB and attP) reaction to generate the *BHD*-5′ and *BHD*-3′ homology entry clones, respectively. A 1.3-kb fragment of genomic DNA bearing exons 3 and 4 of the *BHD* gene was inserted to the modified pDONR vector *pENTR3C-loxPMCS-loxP-FRT-neo-FRT* between the *Sal*I and *Not*I sites to generate a *BHD-exon3-4-pENTR3C* entry clone. The three entry clones, in combination with the modified destination vector, were incubated to create a *BHD-pDESTR4R3* targeting construct through BP recombination reaction. (B) Positive-targeting ES clones were selected by long-range PCR and confirmed by Southern blot analysis. (C) PCR genotyping of mouse offspring using tail DNA. (D, E) Knockout mice and normal controls were validated by Southern blot analysis.

To generate a conditional knockout construct, we adopted the MultiSite Gateway**®** Three-Fragment Vector Construction system (Invitrogen, Carlsbad, CA) to inactivate the *BHD* gene by deleting exons 3 and 4 (**Figure 1A**). The construct was electroporated into 129/Sv strain embryonic stem (ES) cells. Correctly targeted ES cell clones were obtained after being selected with G418, screened by long-range PCR, and confirmed using PCR and Southern blot analysis (**Figure 1B–E**). For the generation of chimeras, ES cells heterozygous for the *BHD*-floxed allele were injected into C57BL/6 blastocysts by standard procedures. Chimeras were bred to C57BL/6 mice to produce *BHD^flox/+^* heterozygotes, and germ-line offspring were identified by PCR genotyping (**Figure 1C**).

### *BHD* null mice are embryonic lethal

To determine whether ablation of *BHD* affected the viability of mice, we first generated a conventional *BHD*-deficient mouse model by intercrossing *BHD^flox/flox^* mice with *CMV-Cre* transgenic strains that express Cre recombinase in all tissues. While most heterozygous *BHD^+/−^*/*CMV-Cre* mice showed no obvious abnormalities at age of 18 months, the homozygous mutation was embryonic lethal and *BHD* ^−/−^ mutants died between 3.5 dpc and 8.5 dpc, underscoring the importance of *BHD* in development. Indeed, genes that are important in embryonic development are frequently found to be the culprits in human cancers.

### Kidney-specific inactivation of *bhd* results in renal cysts

**Figure 2:**
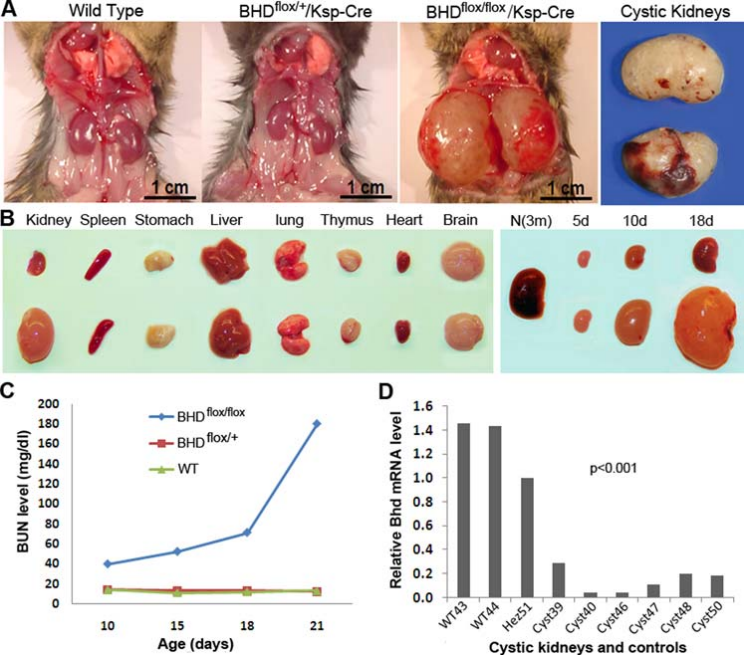
Phenotypes of *BHD^flox/flox^/Ksp-Cre* mice. (A) *BHD^flox/flox^/Ksp-Cre* mice developed polycystic kidney and died at age of three weeks. The kidneys from heterozygotes were phenotypically normal, similar to the ones from wild-type mice. (B) The organs from the affected mice were normal except for the kidneys (10 days old). There was not much size difference between the normal control kidneys and the affected kidneys at birth. However, the difference became apparent after the age of 5 days. At age of 10 days, the polycystic kidneys were approximate 15 times larger than those from normal controls. (C) Biochemical analysis revealed that the conditional knockout mice died of kidney failure due to high level of blood urea nitrogen (BUN). The BUN level dramatically elevated 15 days after birth. Most of the mice died at the age of 21 days. (D) The mRNA level of *BHD* in the kidneys of *BHD^flox/flox^/Ksp-Cre* mice is significantly lower than that in wild-type kidneys. The heterozygote also shows lower mRNA expression relative to the wild type. m = month; d = day. f/f = flox/flox; f/+ = flox/+.

**Figure 3:**
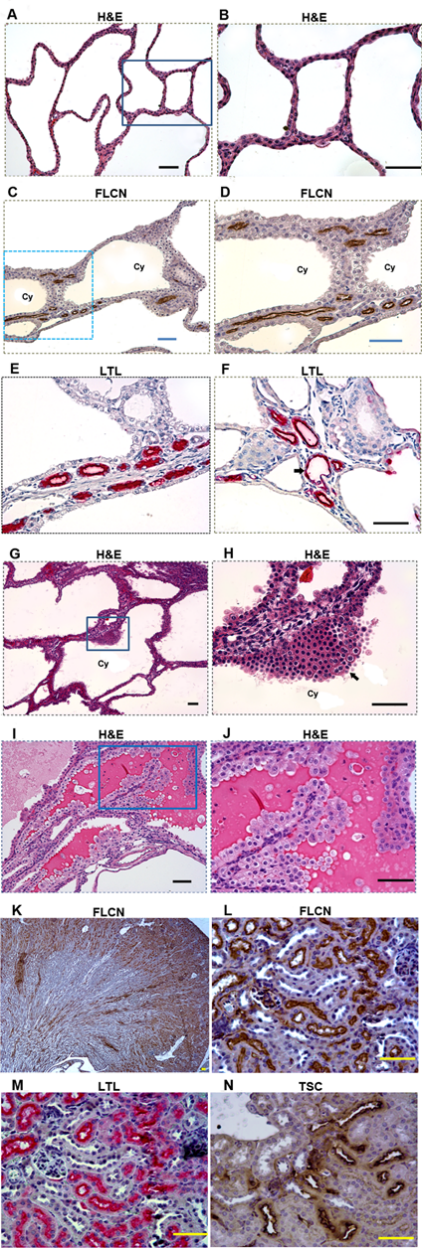
Inactivation of FLCN in *BHD^flox/flox^/Ksp-Cre* mice led to polycystic disease, hyperplasia, cystic renal cell RCC. (A,B) Deficiency of FLCN resulted in polycystic disease. (C, D) No FLCN expression was detected in cysts (enlarged tubules). However, FLCN was still expressed in relatively normal tubules where *BHD* was not deleted or completely eliminated by Ksp-Cre due to no or low Ksp-Cre expression in some proximal tubules. (E, F) Most of the relative normal tubules were proximal tubules stained by proximal tubule-specific marker lotus tetragonolobus lectin (LTL). Many of the proximal tubules remained relatively normal, though some proximal tubules were also enlarged (indicated by arrow. (G, H) Hyperplasia (indicated by arrow) was frequently observed in the cysts. (I, J) Cystic renal cell carcinomas were also one of the important consequences of kidney-targeted *BHD* gene inactivation in *BHD^flox/flox^/Ksp-Cre* mice, which is morphologically distinct from regular cysts showed in A and B. (K, L) FLCN is predominately expressed in proximal tubules, which was demonstrated by the proximal-specific marker LTL (M). FLCN expression is quite weak in distal tubules, which was marked by the distal-tubule-specific marker, Na-Cl-cotransporter (TSC) (N). Scale bar = 50 µm.

BHD patients have a strong predisposition to develop bilateral and multifocal renal tumors with a variety of histologies [22], implying an effect of *BHD* on kidney tumorigenesis. We thus generated a kidney-specific knockout by breeding *BHD^flox/flox^* mice to *Ksp-Cre* transgenic mice with expression of Cre-recombinase under the control of the kidney-specific cadherin promoter [23]. While the *BHD* ^flox/+^/*Ksp-Cre* heterozygous mice showed a normal phenotype at the age of 18 months, the homozygous *BHD^flox/flox^/ Ksp-Cre* mice developed bilateral polycystic kidneys that were over tenfold heavier than those of *BHD^flox/+^*/*Ksp-Cre* and wild-type littermate controls (**Figure 2A,B**). The *BHD* ^flox/flox^/*Ksp-Cre* mice died of kidney failure at the age of 3 weeks, having over 10 times higher levels of blood urea nitrogen (BUN) than normal littermate controls (**Figure 2C**). The considerably low levels of BHD mRNA detected by real time RT-PCR demonstrated inactivation of *BHD* in most of the kidney cells (**Figure 2D**). The appearance of the cysts here is similar to that found in polycystic kidney disease caused by mutated *PKD* genes (**Figure 3A,B**). Histopathological examination of the *BHD^flox/flox^*/*Ksp-Cre* kidneys revealed extremely dilated renal tubules that predominantly originated from collecting ducts due to high expression of Ksp-Cre recombinase. While some proximal tubules were highly or moderately dilated, most of the other proximal tubules remained relatively normal as a result of extremely low expression levels of Ksp-cre recombinase (**Figure 3C–F**). Atrophic, compressed glomeruli were also observed, and degeneration, necrosis, and haemorrhage were frequently observed in the late stages. These morphological changes suggest that homozygous *BHD* inactivation in the kidney may cause loss of growth control in tubular epithelial cells.

### Kidney-specific inactivation of *bhd* produced renal cell carcinoma (RCC)

We further examined whether *BHD^flox/flox^/ Ksp-cre* mice develop renal carcinomas along with the cysts. We observed that kidneys from mice less than two weeks old predominately presented dilated tubules and cysts, whereas mice more than 18 days old also developed hyperplasia and renal cell carcinoma in their polycystic kidneys (**Figure 3G–J**). Hyperplastic areas frequently exhibited as multiple layers of epithelial cells along the inner surface of the tubules (**Figure 3G, H**). Renal cell carcinoma, which presents as cystic RCC, was frequently observed in the extremely enlarged kidneys. Cystic RCC was first described in 1986 and more cases have been reported since then [24], [25], [26], [27], [28], [29], [30], [31], [32]. Images of human cystic RCC are also available in the webpathology website (http://www.webpathology.com/image.aspcase66n8; http://www.webpathology.com/image.aspcase66n9). The occurrence of cystic RCC in the general population is 4 to 10%, or 1 to 2% of all renal tumors. The cystic RCC does not present as a solid mass, but rather as a unilocular or multilocular cystic mass that is composed of cancer cells growing in the form of cysts that are distinct from regular cysts (**Figures 3I,J** **and** **S1**). While some of the tumor cells lined the septa, the others protruded into the cystic lumen. Most of the tumor cells were larger than the regular cystic cells. Binucleated cystic RCC cells were also observed. Many cystic spaces are filled with hemorrhage or proteinaceous fluid. No solid tumors were observed in any of the affected mice, which may be attributed to their short lifespan; three weeks might not be sufficient for solid tumor development.

### Deficiency of FLCN and subsequent activation of mTOR contributed to renal cysts and RCCs

To elucidate the biochemical mechanisms of the cystogenesis and carcinogenesis related to inactivation of the *BHD* gene, we investigated the possible relevance of *BHD* to the mTOR signaling pathway for the following reasons: 1) our microarray analysis revealed that ectopic expression of the *BHD* gene product, FLCN, led to down-regulation of the AKT- related mTOR pathway signature (**Figure S2**); 2) *BHD*, *PTEN*, *LKB1*, and *TSC1/2* are all hamartoma syndrome–related genes, and the roles of PTEN, LKB1, and TSC1/2 in the mTOR pathway have been well-established; and 3) *in vitro* experiments indicated that FLCN interacted with AMPK, a member of the mTOR pathway [18]. All these clues implied that *BHD* gene may play an important role in suppression of cystogenesis and tumorigenesis and that its inactivation could lead to the formation of renal cysts and RCC through the mTOR pathway.

Before investigating the correlation of FLCN with mTOR pathway, we first examined the distribution of FLCN in normal mouse kidney and polycystic kidney. To do this, we designed and developed a human BHD monoclonal antibody that is compatible with immunohistochemical analysis in the mouse. While FLCN was predominantly expressed in the normal proximal tubules and collecting ducts in the cortex, obvious expression was rarely observed in the kidney distal tubules of mice at age of 3 weeks (**Figure 3K–N**). In the polycystic kidney, FLCN was only detected in relatively normal tubules (**Figure 3C–F**), which are mainly proximal tubules. A small number of proximal tubules were also enlarged due to moderate expression of Ksp-Cre recombinase (**Figure 3F**, arrow), which is different from the previous report where the proximal tubules are not involved. All the enlarged tubules were FLCN-negative (**Figure 3C,D**), indicating a correlation of the formation of cysts with inactivation of the *BHD* gene.

**Figure 4:**
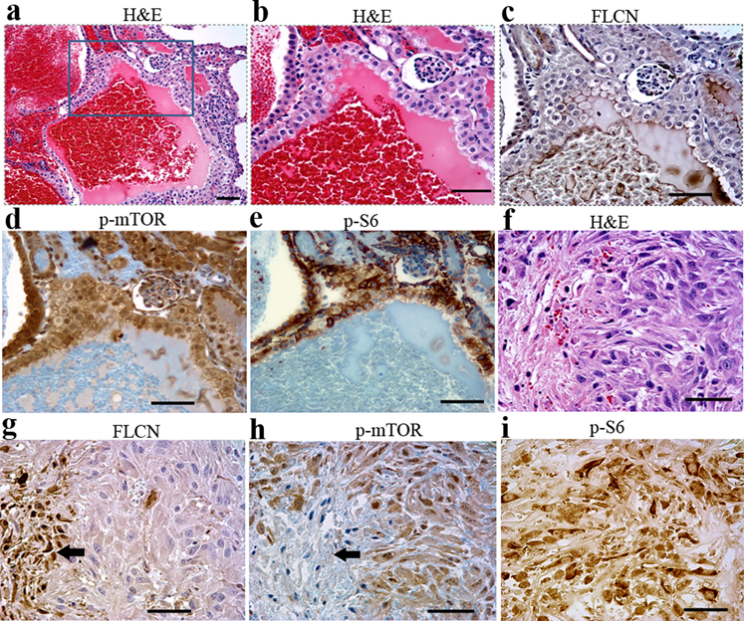
mTOR signaling pathway was activated in the cystic cells, cystic RCC cells. (A) Cystic RCC was stained by hematoxylin and eosin (H&E). (B) No FLCN expression was detected in cystic RCC, indicating deletion of the *BHD* gene. Phosphorylated mTOR (C) and phosphorylated S6 (D) staining was observed in the corresponding FLCN-deficient cells. Scale bar = 50 µm.

We then explored whether the inactivation of *BHD* resulted in the activation of mTOR in affected cysts and RCCs. Immunohistochemical analysis showed that mTOR was activated through phosphorylation in cysts and cystic RCCs (**Figure 4A–C**), which stained FLCN-negative (**Figure 4B**). We further examined the phosphorylation status of the downstream target S6 (**Figure 4D**). Phosphorylated S6 has been observed in some cysts and in cystic RCC. Although FLCN was reported to be a possible downstream effector of mTOR in an *in vitro* experiment[18], our data revealed that deficiency of FLCN activated mTOR pathway *in vivo*, suggesting mTOR might a downstream target of FLCN. To further elucidate the correlation of FLCN and mTOR, we applied the mTOR inhibitor rapamycin to affected mice to see whether we could inhibit or reverse the development of cysts. Rapamycin treatment significantly extended the survival period of *BHD^flox/flox^*/*Ksp-Cre* mice and inhibited the development of cysts relative to control mice; some mice survived more than 50 days. However, once the rapamycin treatment was stopped, cysts redeveloped rapidly and the mice died within 10 days. This result indicated that rapamycin can inhibit cystic cell growth, but cannot reverse the cystic kidney phenotype. We also tested a few other members of the mTOR pathway (e.g. AKT) through IHC; no significant changes were observed or inconsistent results were obtained following inactivation of *BHD*, implying a novel FLCN-mTOR pathway branch may exists. In addition, FLCN might be related to other signaling pathways. Obviously, the precise *in vivo* mode of action of FLCN merits more investigation.

## Discussion

In this study, we provide the first evidence that the *BHD* protein FLCN predominantly expresses in the proximal tubules and collecting ducts of the renal cortex (**Figure 3K–N**). By developing and subsequently analyzing the conditional BHD knockout mouse model, we demonstrate that the deletion of *BHD* in the mouse kidney leads to cystic renal cell carcinoma (cystic RCC) in addition to polycystic kidney and hyperplasia. The cystic RCC was only observed in the older affected mice (≥20 days old). This implies that most of the polycystic kidneys would only present regular cysts and various extents of hyperplasia if the affected mice are sacrificed earlier. Thus, although some kidney-specific knockout animal models of RCC-related genes failed to develop RCC[21], [33], [34], our data provide a connection between kidney-specific *BHD* gene inactivation and renal carcinogenesis. This finding suggests that BHD may act as a suppressor for both cystogenesis and tumorigenesis.

No solid kidney tumors were observed in any of the affected mice, which may be attributed to their short lifespan and mouse distinct genetic background. It is entirely possible that if the cysts had not caused kidney failure at age of three weeks, progression of these cystic RCC to solid tumors would have occurred. In addition, inactivation of *BHD* gene in the kidney causes a large proportion of tubules to form cysts. Once cystogenesis starts, fast-growing cysts become dominant and lead to highly cystic kidneys, kidney failure, and early death. Thus, lack of appropriate microenvironment might be another reason that the malignant/pre-malignant cells failed to form solid renal tumors, which is a more complicated and slower process.

Our results further demonstrated that deficiency of *BHD* product FLCN led to activation of mTOR pathway in cystic cells, supporting the recent report and consolidating that FLCN is involved in mTOR pathway and mTOR may be downstream target of FLCN[21]. Interestingly, BHD is a member of the hamartoma syndrome family that includes Cowden syndrome (CD, affected gene *PTEN*), Peutz-Jeghers syndrome (PJS, affected gene *LKB1*), and tuberous sclerosis complex (affected genes *TSC1/TSC2*) [14], [15], [16]. While PTEN, LKB1, and TSC1/2 have played pivotal roles in the mTOR pathway, our findings suggest that *BHD* protein FLCN, like other hamartoma syndrome–related proteins such as PTEN, LKB1, and TSC1/2, is an important component of the mTOR pathway, constituting a novel FLCN-mTOR signaling branch that regulates cell growth/proliferation, though FLCN may involve in other pathways.

## Materials and Methods

### Design and generation of *BHD* conditional knockout construct

The MultiSite Gateway**®** Three-Fragment Vector Construction system (Invitrogen, Carlsbad, CA) was modified for the purpose of fabricating recombination vectors [35]. Of the four vectors supplied in the system, the pDONR vectors, pDONR P4-P1R, and pDONR P2R-P3 were used to generate the 5′ and 3′ homology arm entry clones. Another vector, pENTR3C, was used to carry a targeted gene sequence of interest. To meet the gene targeting purpose, a 1.8-kb loxP-FRT-neo-FRT fragment excised from p-loxp-2FRTPGKneo (a gift of D. Gordon) was added to generate pENTR3CloxP-FRT-neo-FRT, which allowed later excision of *BHD* exons 3 and 4 and the neomycin-resistance gene by cre-mediated recombination *in vivo*. Synthetic oligonucleotides were used to insert an additional *loxP* site into the *Dra*I site of the pENTR3C-loxPFRT- neo-FRT vector. Oligonucleotides loxPF (5′-ATAACTTCGTATAGCATACATTATACGAAGTTATTT-3′) and loxPR1 (5′-AAATAACTTCGTATAATGTATGCTATACGAAGTTAT-3′) (IDT, Coralville, IA) were phosphorylated with T4 polynucleotide kinase (Invitrogen), annealed, and inserted into the *Dra*I-digested pENTR3C-loxP-FRTneo-FRT vector to generate pENTR3C-loxPMCS-loxP-FRT-neo-FRT.

To enrich targeted ES cell clones, we inserted a TK-negative selection cassette downstream of the attR3 site in the destination vector. The attR4-*ccdB*-attR3 domain was amplified from the pDEST/R4-R3 vector using the following primers: 5′-GCCTCGAGCAGGAAACAGCTATGAC-3′ and 5′-GCCTCGAGTAAAACGACGGCCAGTG-3′. After digestion by *Xho*I, this domain was inserted into the *Xho*I site of the pPGKneo/TK vector (a gift from T. Gridley).

To generate a *BHD* gene targeting construct, a 3.5-kb 5′ homology arm containing exon 2 and a 3.0-kb 3′ arm carrying exons 5 and 6, PCR-amplified using *Pfx* polymerase (Invitrogen), were integrated into the pDONR P4-P1R and pDONR P2R-P through BP reaction (attB and attP sites) to generate the BHD-5′ and BHD-3′ homology entry clones, respectively. A 1.3-kb fragment of genomic DNA bearing exons 3 and 4 of the *BHD* gene was inserted into the modified pDONR vector pENTR3C-loxPMCS-loxP-FRT-neo-FRT between the *Sal*I and *Not*I sites to generate a BHD-exon3-4-pENTR3C entry clone. Finally, the three entry clones, in combination with the modified destination vector, were incubated to create a BHD-pDESTR4R3 targeting construct through BP recombination reaction.

### Identification of homologous recombinant ES cells and generation of kidney-specific knockout mice

The generated BHD-pDESTR4R3 targeting construct carries an ampicillin-resistant gene and a neomycin-resistant gene flanked by FRT sites. The construct was linearized with *Sca*I for electroporation into 129/sj strain ES cells. After selection with 500 µg/ml G418 (Invitrogen), 1,039 ES cell clones were isolated. The G418-positive ES clones were first screened by long-range PCR and then confirmed by Southern blot analysis. For the generation of chimeras, ES cells heterozygous for the *BHD^flox/+^* allele were injected into C57BL/6 blastocysts by standard procedures. Chimeras were bred to C57BL/6 mice, and germline offspring were identified by PCR genotyping. To remove the neomycin gene flanked by two FTR sites, *BHD^flox/+^* mice were crossed to FlpeR transgenic mice that express the site-specific recombinase FLP (FLPe). Then, *BHD^flox/+^* heterozygous mice were intercrossed to give rise to mice homozygous for the *BHD^flox^* allele, i.e., *BHD^flox/flox^* mice.

To obtain mice with kidney-specific inactivation of *BHD*, *BHD^flox/flox^* mice were first bred to Ksp-Cre transgenic mice to generate *BHD* heterozygous mice (*BHD^flox/+^/Ksp-Cre* in kidneys). *BHD^flox/+^/Ksp-Cre* mice then were backcrossed to *BHD^flox/flox^* mice to generate *BHD* homozygous mice (*BHD^flox/flox^/Ksp-Cre* in kidneys).

All mice were manipulated and housed according to protocols approved by the Institutional Animal Care and Use Committee (IACUC) of Van Andel Institute and conducted in an ethical, humane, and scientifically justified manner, and in full compliance with applicable regulations.

### Genotyping, RNA and protein analysis

ES cell DNA and tail DNA was extracted by using automated DNA isolation system (Kurabo Industries) and subjected to regular PCR and long-range PCR genotyping analysis (see supplementary Table S1 for PCR primers). For genotyping by Southern blot analysis, DNA from ES cells or tissues was extracted using standard DNA extraction procedure. Purified DNA was digested by *Xmn* I or *Hin*d III, isolated by 0.8% agarose gel, and transferred onto nylon membrane. UV-linked or dried membranes were subjected to DNA hybridization with 5′ or 3′ probes.

Total RNA was isolated from various mouse tissues and cystic cell lines with Trizol reagent (Invitrogen) according to the manufacturer's instructions. Purified RNA was used for quantitative analysis (real-time RT-PCR) through ABI Prism 7700 Sequences Detector (Applied Biosystems).

For protein detection by Western blot, cultured cells and kidney whole-cell extracts prepared by homogenization were lysed in 1% Nonidet P-40, 50 mM Tris (pH 7.4), 150 mM NaCl, 1 mM EDTA, and 15% glycerol, plus standard protease inhibitors (protease inhibitor cocktail tablets, Roche Diagnostics). Equal amounts of mutant and control kidney cell protein extracts were size-separated by 10% SDS-PAGE and transferred to PVDF membranes (Invitrogen). FLCN was detected with a mouse monoclonal anti-FLCN antibody (developed by Laboratory of Antibody Technology) at a dilution of 1∶750 using the enhanced chemiluminescence detection system.

### Immunohistochemistry and tubular marker staining

Immunohistochemical analysis was performed following the manufactory's protocols. The antibodies used include anti-FLCN mAb, anti-Phospho-mTOR Rabbit mAb (Cell Signaling), anti-Phospho-S6 Ribosomal Protein (Cell Signaling). Proximal tubules were stained by biotinylated Lotus Tetragonolobus Lectin (LTL, Vector Laboratories), and distal tubules were detected by using rabbit anti-thiazide-sensitive NaCl contransporter affinity purified polyclonal antibody (TSC, Chemicon) Tubular markers. Marker biotinylated Peanut Agglutinin (PNA, Vector Laboratories) was used to stain collecting ducts.

### Phenotyping and histopathology

Newborn mice were monitored daily. Sick mice were distinguished from healthy ones by enlarged abdomen at age of 10 days. Totally 73 *BHD^flox/flox^/Ksp-Cre* mice and 55 normal control littermates were collected for phenotyping and histopathological analysis. Mouse body weight, kidney weight were measured upon euthanasia by CO_2_ inhalation. Tissues including kidneys, lung, liver, spleen, heart, stomach, intestine, brain, testes were collected and fixed in 4% paraformaldehyde for 24 hours and kept in 70% ethanol for 12 hours before paraffin block preparation. Paraffin blocks were sectioned at 3 µm thick and stained with hematoxylin and eosin (H&E). Stained slides were evaluated by a board-certified veterinary pathologist B. Sigler and pathologists X. Yang and J. Peng.

### Blood biochemical analysis

Mouse blood was collected by cardiac puncture at age of 5 days, 10 days, and 15 days, and 20 days. Serum was collected after centrifugation and stored at −80°C for further biochemical analysis.

### Rapamycin treatment of *BHD^flox/flox^/Ksp-Cre* mice and *BHD^flox/+^/Ksp-Cre* and wild-type control littermates

Totally 29 mice from three litters were used for rapamycin treatment (n = 15) and control (n = 14). Mice from each litter at postnatal 7 days were randomly divided into two groups of rapamycin treatment and control. Rapamycin (LC Laboratories, Woburn, MA) was dissolved in ethanol at a concentration of 20 mg/mL and stored at −20°C. The rapamycin solution was freshly prepared by diluting the rapamycin stock to 250 µg/ml in buffer (1∶1 10% PEG-400, 8% ethanol:10% Tween 80) and were injected by intraperitoneally daily at a dose of 2.5 mg/kg body weight for the duration of the treatment. Control animals received i.p. injection of equal amount of vehicle (5% PEG-400, 4% ethanol, and 5% Tween 80). Mice were treated for at least two weeks starting at postnatal day 7. Moribund mice were subjected to CO_2_ euthanasia, dissection, and analysis in the duration of the treatment.

## Supporting Information

Table S1 Primer used for BHD knockout genotyping
(0.02 MB XLS)

Figure S1 Additional cystic RCC samples stained by hematoxylin and eosin. Cystic spaces are filled with proteinaceous fluid (A–J) or hemorrhage (K,L) in cystic RCC. The tumor cells have clear cytoplasm and hyperchromatic nuclei lining the septa or growing into the cystic lumina. Scale bar = 50 µm.
(5.38 MB TIF)

Figure S2 Microarray analysis revealed that ectopic expression of FLCN led to down-regulation of the AKT- related mTOR pathway signature.
(0.29 MB TIF)
