# Correction: Deficiency of FLCN in Mouse Kidney Led to Development of Polycystic Kidneys and Renal Neoplasia

**DOI:** 10.1371/annotation/0f06471c-1993-4144-b785-d58924ac0b5c

**Published:** 2008-11-19

**Authors:** Jindong Chen, Kunihiko Futami, David Petillo, Jun Peng, Pengfei Wang, Jared Knol, Yan Li, Sok-Kean Khoo, Dan Huang, Chao-Nan Qian, Ping Zhao, Karl Dykema, Racheal Zhang, Brian Cao, Ximing J. Yang, Kyle Furge, Bart O. Williams, Bin Tean Teh

The published Figure 4 is an incorrect version, including too many panels. Please view the correct figure, containing only panels (A-D), here:

**Figure 4:**
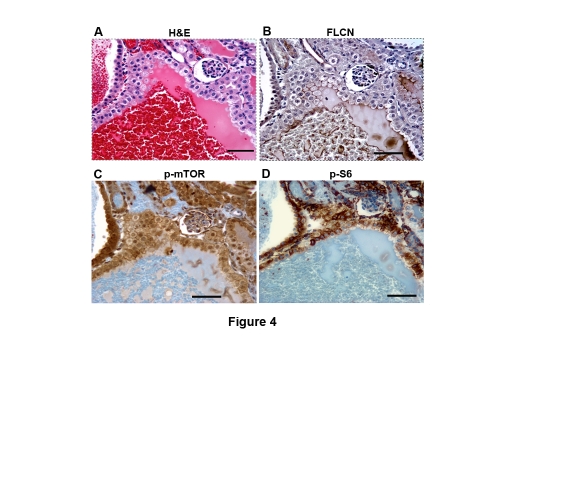
mTOR signaling pathway was activated in the cystic cells, cystic RCC cells. (A) Cystic RCC was stained by hematoxylin and eosin (H&E). (B) No FLCN expression was detected in cystic RCC, indicating deletion of the *BHD* gene. Phosphorylated mTOR (C) and phosphorylated S6 (D) staining was observed in the corresponding FLCN-deficient cells. Scale bar = 50 µm.

