# Correction: Deficiency of FLCN in Mouse Kidney Led to Development of Polycystic Kidneys and Renal Neoplasia

**DOI:** 10.1371/annotation/136385d5-b241-4ecc-b31a-6dea3ebf3bc4

**Published:** 2008-11-19

**Authors:** Jindong Chen, Kunihiko Futami, David Petillo, Jun Peng, Pengfei Wang, Jared Knol, Yan Li, Sok-Kean Khoo, Dan Huang, Chao-Nan Qian, Ping Zhao, Karl Dykema, Racheal Zhang, Brian Cao, Ximing J. Yang, Kyle Furge, Bart O. Williams, Bin Tean Teh

The 12th author's name was spelled incorrectly. It should read: Karl Dykema. 

