# Supplementary figures and images for: Deficiency of FLCN in Mouse Kidney Led to Development of Polycystic Kidneys and Renal Neoplasia
Source: PLoS One. 2008 Oct 30;3(10):e3581. doi: 10.1371/journal.pone.0003581 (PMC2570491; doi:10.1371/journal.pone.0003581)

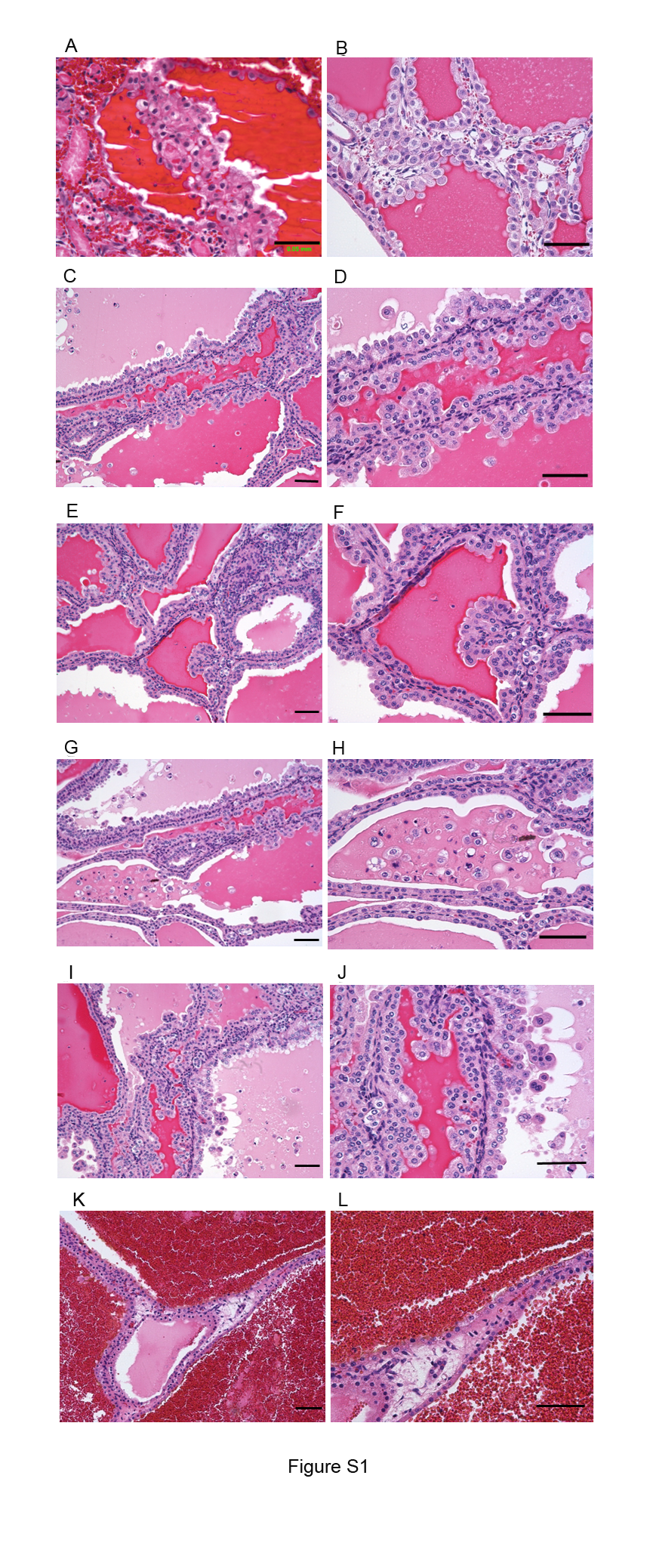

Supplement: Figure S1 — Additional cystic RCC samples stained by hematoxylin and eosin. Cystic spaces are filled with proteinaceous fluid (A–J) or hemorrhage (K,L) in cystic RCC. The tumor cells have clear cytoplasm and hyperchromatic nuclei lining the septa or growing into the cystic lumina. Scale bar = 50 µm. (5.38 MB TIF) [file pone.0003581.s002.tif]

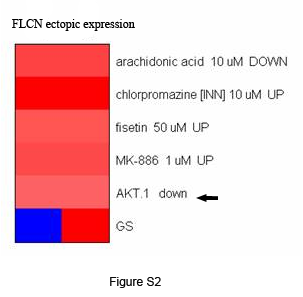

Supplement: Figure S2 — Microarray analysis revealed that ectopic expression of FLCN led to down-regulation of the AKT- related mTOR pathway signature. (0.29 MB TIF) [file pone.0003581.s003.tif]
